# An Autophagy-Associated MITF–GAS5–miR-23 Loop Attenuates Vascular Oxidative and Inflammatory Damage in Sepsis

**DOI:** 10.3390/biomedicines11071811

**Published:** 2023-06-24

**Authors:** Junning Cheng, Chang Ding, Huying Tang, Haonan Zhou, Mingdong Wu, Yikuan Chen

**Affiliations:** 1 Department of Vascular Surgery, The Second Affiliated Hospital of Chongqing Medical University, Chongqing 400010, China; cjnvsd@yeah.net (J.C.); cqmuvascular@163.com (H.Z.); wmdcqmu@yeah.net (M.W.); 2 Central Laboratory of School of Life Sciences, Chongqing Medical University, Chongqing 400016, China; dingccecho@foxmail.com (C.D.); lyaansel@stu.cqmu.edu.cn (H.T.); 3 Department of Ultrasound, Chongqing Traditional Chinese Medicine Hospital, Chongqing 400013, China; 4 Department of Radiology, Southwest Hospital, Third Military Medical University (Army Military Medical University), Chongqing 400038, China

**Keywords:** sepsis, oxidative stress, inflammation, MiT–TFE transcription factor, autophagy

## Abstract

Background: Sepsis induces GAS5 expression in the vascular endothelium, but the molecular mechanism is unclear, as is the role of GAS5 in sepsis. Methods and results: We observed that GAS5 expression in the endothelium was significantly upregulated in a sepsis mouse model. ChIP-PCR and EMSA confirmed that the oxidative stress (OS)-activated MiT–TFE transcription factor (MITF, TFE3, and TFEB)-mediated GAS5 transcription. In vitro, GAS5 overexpression attenuated OS and inflammation in endothelial cells (ECs) while maintaining the structural and functional integrity of mitochondria. In vivo, GAS5 reduced tissue ROS levels, maintained vascular barrier function to reduce leakage, and ultimately attenuated sepsis-induced lung injury. Luciferase reporter assays revealed that GAS5 protected MITF from degradation by sponging miR-23, thereby forming a positive feedback loop consisting of MITF, GAS5, and miR-23. Despite the fact that the OS-activated MITF–GAS5–miR-23 loop boosted MITF-mediated p62 transcription, ECs do not need to increase mitophagy to exert mitochondrial quality control since MITF-mediated Nrf2 transcription exists. Compared to mitophagy, MITF-transcribed p62 prefers to facilitate the autophagic degradation of Keap1 through a direct interaction, thereby relieving the inhibition of Nrf2 by Keap1, indicating that MITF can upregulate Nrf2 at both the transcriptional and posttranscriptional levels. Following this, ChIP-PCR demonstrated that Nrf2 can also transcribe MITF, revealing that there is a reciprocal positive regulatory association between MITF and Nrf2. Conclusion: In sepsis, the ROS-activated MITF–GAS5–miR-23 loop integrated the antioxidant and autophagy systems through MITF-mediated transcription of Nrf2 and p62, which dynamically regulate the level and type of autophagy, as well as exert antioxidant and anti-inflammatory effects.

## 1. Introduction

Sepsis, defined as “life-threatening organ dysfunction due to a dysregulated host response to infection”, is a common condition with high morbidity and mortality rates worldwide [1]. In sepsis, dysregulated inflammation and oxidative stress (OS) can rapidly lead to multiorgan failure, primarily affecting the cardiovascular and respiratory systems [2,3,4]. The lung is the most vulnerable organ in sepsis, and lung injury is initially characterized by vascular fluid leakage through paracellular pathways, clinically manifesting as acute respiratory distress syndrome (ARDS). Sepsis causes an imbalance between the levels of reactive oxygen species (ROS) and antioxidant agents in endothelial cells (ECs), which, in addition to mitochondrial dysfunction, exacerbates the dysfunction of ECs in multiple ways, such as glycocalyx breakdown, EC apoptosis, and junction protein remodeling [5]. Since ECs serve as both a source and a target of oxidative stress during sepsis, severe vascular leakage caused by endothelial injury is currently regarded as one of the most important pathophysiological changes during septic lung injury [6].

Under physiological and many pathophysiological conditions, mitochondria are the primary site of ROS production from aerobic respiration [7]. Mitochondrial dysfunction is currently regarded as a significant factor in sepsis-induced organ failure, potentially impairing cellular energy supply and increasing OS [8]. In sepsis, autophagy, a catabolic process that recycles cellular components and damaged organelles in response to various stressors such as oxidative stress, is commonly viewed as a protective adaptive mechanism that limits cellular damage and apoptosis [9,10,11]. Mitophagy is the specific autophagic response that targets and removes cytotoxic mitochondria to avoid further damage [8,12]. Upon mitochondrial damage and reduced membrane potential (Δψm) caused by ROS overproduction, the mitochondrial kinase PINK1 and the E3 ubiquitin ligase Parkin cooperate to ubiquitinate outer membrane components, initiating autophagosome formation through the adaptor protein p62-mediated LC3 binding [12,13,14]. Among the adaptor proteins, p62 plays a crucial role not only in mitophagy, but also in impeding the Keap1-mediated degradation of Nrf2 by directly engaging with Keap1 [15,16,17,18]. The activation of the antioxidant Keap1–Nrf2–ARE pathway is vital for maintaining mitochondrial structural and functional integrity under stress, as it regulates antioxidant response element (ARE)-dependent genes, including SQSTM1 (p62), and establishes a p62–Keap1–Nrf2 positive feedback axis for persistent Nrf2 activation and p62 replenishment [19,20].

Long noncoding RNAs (lncRNAs) play an important role in regulating vascular homeostasis, as previously demonstrated [21]. The lncRNA, growth arrest-specific 5 (GAS5), has increasingly been recognized as a key modulator in sepsis-induced organ injury. In specific instances such as lipopolysaccharide (LPS)-induced myocardial cell damage, GAS5 has demonstrated potential in attenuating myocardial cell apoptosis, indicating a protective role [22]. Nonetheless, the landscape of the available literature also includes a study positing that GAS5 may amplify myocardial injury and an inflammatory response under septic conditions [23], which underscores the complexity of its role. In relation to septic kidney injury, emerging data suggest that GAS5 may serve as a protective mechanism, potentially through its ability to reduce cell pyroptosis [24]. Additionally, GAS5 has been implicated in mitigating inflammatory response and apoptosis in both alveolar and bronchial epithelial cells [25,26]. This illustrates another dimension of GAS5’s role in organ-specific responses to sepsis. While the role of GAS5 in sepsis-induced organ injury has been increasingly acknowledged, the existing literature seems to slightly overlook the exploration of the upstream regulatory mechanisms that induce GAS5 expression. A specific area that requires additional attention is the impact of GAS5 on vascular endothelial cells under septic conditions. Considering the lung as a primary organ susceptible to sepsis, and vascular leakage being one of its principal injury mechanisms, this indeed highlights the necessity for our intended research in this specific area. Our study shows a significant upregulation of lncRNA GAS5 in the vascular endothelium of septic mice, but the exact mechanism remains unclear. Upon reviewing published studies, we discovered that the expression of GAS5 can be induced by oxidized low-density lipoprotein (Ox-LDL) and the autophagy agonist rapamycin [27,28,29,30,31]. Rapamycin works as an autophagy agonist by inhibiting the function of mTOR complex 1 (mTORC1), which is composed of the mechanical target of rapamycin (mTOR) and multiple related proteins such as Raptor, PRAS40, and DEPTOR. Among them, mTOR is a member of the PI3K-related kinase family and functions as a redox sensor [32,33,34]. When mTOR is dephosphorylated, the function of mTORC1 is inhibited, rendering mTORC1 incapable of preventing the nuclear localization of the transcription factor TFEB [35], a member of the MiT–TFE family (TFEB, TFEC, TFE3, and MITF), which is an important regulator of lysosome biogenesis and autophagy [36].

In this study, we found that GAS5 expression is regulated by the MiT–TFE family of transcription factors. Our examination of the effect of GAS5 revealed that it can inhibit oxidative stress and maintain mitochondrial homeostasis, ultimately leading to the attenuation of lung injury in sepsis. Through the inhibition of the MAPK and PKC signaling pathways, DGKE relieves the proinflammatory and prothrombotic state of ECs [37,38], and we demonstrated that the anti-inflammatory effect of GAS5 is achieved through the GAS5–miR-23–DGKE axis. Overall, GAS5, functioning as an antioxidant and anti-inflammatory lncRNA, may provide a promising target in sepsis.

## 2. Materials and Methods

### 2.1. Animals

All experiments were conducted on male C57BL/6J mice that were 8 to 10 weeks old and weighed between 24 and 27 g. The mice were obtained from the animal center of Chongqing Medical University. This research followed the National Institutes of Health’s Guide for the Care and Use of Laboratory Animals. The protocols were approved by the Institutional Animal Care and Use Committee (IACUC) at The Second Affiliated Hospital of Chongqing Medical University. Prior to conducting the study, ethical approval was received.

### 2.2. In Vivo AAV–ENT-Mediated Endothelial-Specific Gene Overexpression

In vivo endothelial-specific gene expression was achieved through ENT serotype-associated adeno-associated viral (AAV) vectors. Recombinant AAV–ENT vectors carrying GAS5, MITF, DGKE, mmu–miR-23a, a GAS5–shRNA sequence, or an empty vector with a plasmid that had an intercellular adhesion molecule 2 (ICAM2p) promoter were manufactured by the Shanghai GeneChem Company Limited. The virus titer was 10^12^ vg/mL. AAV–ENT–ICAM2p–empty served as a negative control. AAV–ENT–ICAM2p–target gene/empty vector was delivered via tail vein injection (1 × 10^11^ vector genome/mice) combined with an intratracheal injection (5 × 10^10^ vector genome/mice) to achieve pulmonary and systemic vascular endothelium gene transduction. The C57BL/6J mice were placed in a gas anesthesia device and given oxygen and 2.5% isoflurane to induce anesthesia. After confirming that the effect of anesthesia was reliable, the skin of the anterior cervical region of the mice was incised to expose the trachea. Using an insulin syringe, 50 µL of the diluted virus was withdrawn, the needle was inserted into the trachea at a 45-degree angle, the virus was slowly injected, and then the needle was pulled out. Next, an insulin syringe was used to inject 100 µL of the diluted virus into the tail vein of the mice. During the whole operation, the anesthesia system was connected to the noses of the mice to maintain anesthesia. After the operation, the mice were placed in a warm environment while they recovered. Three weeks after adeno-associated virus infection, gene expression was detected, followed by sepsis modeling.

### 2.3. Cecal Ligation and Puncture (CLP) Sepsis Model

Before model induction, all mice were fasted for 8 h, but allowed to consume water ad libitum. The CLP model was constructed with modifications based on a previously published description. Briefly, mice were fully anesthetized by isoflurane inhalation, fixed on an aseptic operating table, and a 1 cm incision was made in the mid-abdomen to expose the cecum. The external two-thirds of the cecum were ligated and punctured once with a 19-gauge needle. After isolating a small amount of feces from the puncture site, the cecum was returned, and the abdominal incision was closed with sterile 6–0 silk sutures. Except for the ligation and perforation of the cecum, sham-operated animals were subjected to the same surgical procedure. All mice received subcutaneous fluid resuscitation with normal saline (50 mL/kg) at 37 °C after surgery.

### 2.4. Cell Lines and Cultures

Human umbilical vein endothelial cells (HUVECs), HEK-293T cells, and THP-1 cells were obtained from PROCELL Biotechnology (Wuhan, China). HUVECs were cultured in RPMI 1640 medium (HyClone, Logan, UT, USA) supplemented with 10% fetal bovine serum and 1% penicillin-streptomycin. HEK-293T cells were cultured in DMEM (HyClone, Logan, UT, USA) supplemented with 10% fetal bovine serum and 1% penicillin-streptomycin. THP-1 cells were cultured in RPMI 1640 medium with 12% fetal bovine serum, 1% penicillin-streptomycin, and 50 µmol/L β-mercaptoethanol. Throughout the experiments, the cells were maintained at 37 °C in a humidified atmosphere containing 5% CO2 and in the exponential growth phase.

### 2.5. Cell Transfection

Small interfering RNA (siRNA) for GAS5, MITF, TFE3, TFEB, Raptor, DGKE, and its negative controls, as well as miR-23a mimics, miR-23b mimics, mimic NC, miR-23a inhibitor, miR-23b inhibitor, and inhibitor NC, were supplied by GenePharma (Shanghai, China). Overexpression plasmids for GAS5, MITF, TFE3, TFEB, and DGKE were supplied by GeneCreate Biological Engineering Co. (Wuhan, China), and the plasmid pcDNA-3.1 was used as a negative control. All transfections were carried out using Lipofectamine 3000 transfection reagent according to the manufacturer’s protocol (Thermo Fisher, Waltham, MA, USA, L3000075).

### 2.6. RNA Isolation and Quantitative Real-Time PCR

HUVECs were treated with TRIzol reagent (Thermo Fisher, Waltham, MA, USA, 15596026) to extract total RNA. Total RNA isolated from cells was subjected to reverse transcription using a PrimeScript RT Master Mix Kit (TaKaRa, Kyoto, Japan, RR036), and microRNA isolated from cells was subjected to reverse transcription using a Mir-X miRNA First-Strand Synthesis Kit (TaKaRa, Kyoto, Japan, 638315). Relative quantification was performed using 2xSYBR Green qPCR Master Mix (Bimake, Shanghai, China, B21203), and the primers were obtained from Sangon Biotech (Shanghai, China). Quantitative real-time PCR was used to determine the levels of mRNA expression using a Bio-Rad iCycler system (Bio-Rad, Hercules, CA, USA).

### 2.7. Western Blot

RIPA lysis buffer was used to lyse and extract total proteins from HUVECs (Beyotime, Shanghai, China, P0013B). Following quantification with a BCA Protein Assay Kit (Beyotime, Shanghai, China, P0010S), protein samples were separated using SDS-PAGE on a 10% or 12.5% acrylamide gel (EpiZyme, Shanghai, China). After that, the proteins were transferred to polyvinylidene fluoride (PVDF) membranes and blocked for 1 h with 5% BSA. Following overnight incubation with primary antibodies at 4 °C, the membranes were incubated for 1 h at room temperature with HRP-labeled secondary antibodies. The membrane was incubated with a substrate for enhanced chemiluminescence (ECL) (EpiZyme, Shanghai, China).

### 2.8. Cellular Immunofluorescence Staining

After 20 min of paraformaldehyde fixation, the samples were washed three times with PBS. The cells were then permeabilized for 10 min with 0.5% Triton X-100, washed three times with PBS, and blocked with goat serum for 1 h. Cellular samples were incubated with the indicated antibodies overnight at 4 °C, washed with PBS three times, and incubated with corresponding secondary antibodies for 1 h at 37 °C. Then, the samples were rinsed in PBS, counterstained with diamidino phenylindole (DAPI) (Beyotime, Shanghai, China, P0131), and mounted with anti-fading reagent (Solarbio, Shanghai, China). Representative images were visualized under a confocal microscope (Nikon A1R, Tokyo, Japan).

### 2.9. Immunofluorescence Staining of Paraffin Sections

Following quick fixation in 4% paraformaldehyde solution for 24 h, the samples were subjected to gradient dehydration (from 50% to 100% ethanol). After that, the samples were cleared with xylene, fixed in paraffin, and cut into 4 µm thick sections to mount onto slides. After deparaffinization and rehydration, the slides were warmed to 60 °C for 1 h in a dry oven to soften the paraffin. For ten minutes each, the slides were washed in xylene I, xylene II, 100% ethanol, 95% ethanol, 90% ethanol, and 80% ethanol. After washing the slides with deionized water for 5 min, they were heated in an oven at 95–100 °C for 20 min in an antigen retrieval solution. The sections were removed from the oven after antigen retrieval and allowed to cool at room temperature. After three washes with PBS, the tissue the sections were covered with goat serum and incubated at room temperature for 1 h. After blocking, the samples were incubated with the indicated antibodies overnight at 4 °C, washed with PBS three times, and incubated with corresponding secondary antibodies for 1 h at room temperature. After washing with PBS three times, the samples were counterstained with DAPI (Beyotime, Shanghai, P0131) and mounted with anti-fading reagent (Solarbio, China). Representative images were photographed using a confocal microscope (Nikon A1R, Tokyo, Japan).

### 2.10. Tissue Reactive Oxygen Species Detection

Frozen sections were thawed at room temperature, and a histochemical pen was used to draw a circle around the tissue. An autofluorescence quencher was added to the area for 5 min, and then the tissue was rinsed with running water for 10 min. ROS staining solution (SIGMA, Darmstadt, Germany, D7008 1:500) was added dropwise to the circular tissue area and incubated in a dark incubator at 37 °C for 30 min. The nuclei were counterstained with DAPI, and the slides were washed three times in PBS (pH 7.4) while shaking on a destaining shaker for 5 min each time. After the sections were slightly dried, DAPI staining solution was added dropwise to the circular area and incubated at room temperature for 10 min in the dark. The slides were washed three times in PBS (pH 7.4) while shaking on a destaining shaker for 5 min each time. After drying, the sections were mounted with anti-fluorescence quenchers.

### 2.11. Immunochemistry (IHC)

Paraffin sections of the largest cross-sections of lung tissue were deparaffinized, hydrated and incubated with 3% H2O2 for 5–10 min at room temperature to eliminate endogenous peroxidase activity. After washing the sections with PBS, antigen retrieval was performed, and then the specimens were blocked with 5–10% goat serum (diluted in PBS). After 10 min of incubation at room temperature, the serum was removed, and the working concentrations of anti-NRF2, p62, Ly6g, ICAM, and VCAM primary antibody solutions were added dropwise. The sections were incubated with the primary antibodies overnight at 4 °C. After washing with PBS, an appropriate amount of biotin-labeled secondary antibody working solution (Servicebio, Wuhan, China) was added dropwise and incubated at 37 °C for 30 min. The slides were rinsed with PBS again, an appropriate amount of a horseradish peroxidase-labeled streptavidin working solution was added dropwise, and the slides were incubated at 37 °C for 10–30 min. After rinsing with PBS, the color developing agent (DAB) (Servicebio, Wuhan, China) was added and the sections were incubated 3–15 min for color development. Then, they were fully rinsed with tap water, counterstained with hematoxylin, routinely dehydrated, cleared, dried, and mounted. Finally, representative images were photographed under an Olympus 600 microscope (Olympus, Tokyo, Japan).

### 2.12. Histological Analysis

After CLP modelling, mouse lung tissue was fixed in DEPC-treated water-containing 4% paraformaldehyde for 24 h. After paraffin embedding, the largest cross-section of the lung was taken for further tissue sectioning. After sectioning, H&E staining was performed, and the sections were photographed for analysis.

### 2.13. Fluorescence In Situ Hybridization (FISH) and Colocalization

HUVECs were used for the FISH and colocalization assay. Shanghai GenePharma Biotechnology Co. Ltd. (Shanghai, China) designed and synthesized Cy3-labeled miR-23a-3p and miR-23b-3p probes, as well as fluorescein amidite (FAM)-labeled human-GAS5, MITF, and DGKE probes. The probe signals were detected using the Fluorescent In Situ Hybridization Kit (GenePharma Biotechnology, Shanghai, China), in accordance with the manufacturer’s instructions. During the procedure, miR-23a-3p and miR-23b-3p red fluorescent probes were incubated with GAS5, MITF, and DGKE green fluorescent probes. Paraffin sections of mouse lung tissue were used for the tissue FISH assay. The Cy3-labeled mouse GAS5 probe was designed and synthesized by Shanghai GenePharma Biotechnology Co., Ltd. (Shanghai, China). The probe signal was detected using a paraffin section fluorescence in situ hybridization kit (GenePharma Biotechnology, Shanghai, China), according to the manufacturer’s instructions. All photos were taken with a Nikon A1R Laser Scanning confocal microscope (Nikon A1R, Tokyo, Japan).

### 2.14. Mitochondrial ROS Detection

According to the manufacturer’s protocol, mitochondrial ROS levels were detected using a MitoSOX™ Red Mitochondrial Superoxide Indicator (Thermo Fisher, Waltham, MA, USA, M36008) and observed under a confocal microscope (Nikon A1R, Tokyo, Japan).

### 2.15. Dual Luciferase Reporter Gene Assays

PCR was used to amplify the wild-type (Wt) and mutant (Mut) fragments of the MITF, DGKE 3’UTR, and GAS5 sequences containing the predicted miR-23a and miR-23b binding sites. To construct wild-type luciferase reporter gene vectors, the miR-23 target site was PCR-amplified from human genomic DNA, and the DNA fragment was cloned into the XhoI and NotI sites on the 3’ end of the luc2 gene in the REPORT vector. To construct mutant-type luciferase reporter gene vectors, the miR-23 target site was changed by PCR mutagenesis. The Wt-REPORT vector and Mut-REPORT vector were cotransfected into HEK-293T cells along with miR-23a mimics, miR-23b mimics, and negative control mimics using Lipofectamine 3000 reagent (Thermo Fisher, Waltham, MA, USA, L3000075) according to the manufacturer’s protocol. Following 48 h of transfection, the dual-luciferase reporter assay system (Promega, Durham, NC, USA) was used according to the manufacturer’s protocol to determine the luciferase activity of the samples compared with that of the Renilla luciferase positive control.

### 2.16. Chromatin Immunoprecipitation (ChIP)

ChIP was performed using the ChIP assay kit (Millipore, Billerica, MA, USA) according to the manufacturer’s instructions. DNA and protein crosslinking was achieved by incubating the cells for 20 min at 37 °C in 1% formaldehyde solution. After sonication, chromatin was immunoprecipitated overnight with 6 μg of anti-MITF, TFEB, Nrf2, Flag antibodies, or 2 μg of a normal IgG antibody (CST, USA). The genomic regions of GAS5, SQSTM1, MITF, TFE3, and NFE2L2 containing the Nrf2-, MITF-, TFEB-, and TFE3-binding sites were amplified by RT-PCR in 20 μL volumes for 30–35 cycles to determine the appropriate conditions for the PCR products of each region. Primer sequences and antibodies are described below.

### 2.17. Electrophoretic Mobility Shift Assay (EMSA)

EMSAs were performed using an EMSA kit (Viagene, Shanghai, China) according to the manufacturer’s instructions. The oligonucleotide probes containing the MITF-binding sequence from the GAS5, SQSTM1, and NFE2L2 promoters were synthesized and labeled with biotin at the 5′ end. The probes were incubated with the nuclear extract at room temperature for 30 min. The entire reaction mixture was run on a nondenaturing 0.5 × TBE 6% polyacrylamide gel for 1 h at 60 V at 4 °C and then transferred onto Biodyne^®^ B nylon membranes (Pall Corporation). Signals were visualized with ChemiDoc XRS (Bio-Rad, Hercules, CA, USA).

### 2.18. Evaluation of Autophagic Flux

mRFP-GFP-LC3 adenovirus transfection was used to monitor autophagic flux by marking and tracking LC3. The mRFP–GFP–LC3 adenovirus construct was obtained from Hanbio Inc. HUVECs were transfected with mRFP–GFP–LC3 adenovirus for 24 h following the manufacturer’s instructions and then transfected with siRNA or plasmid for 48 h. The cells were then transferred to a confocal dish and treated with H2O2 after the cells adhered. Finally, a confocal microscope was used to observe the cells and acquire images (Nikon A1R, Tokyo, Japan).

### 2.19. Evaluation of Mitophagy

A mitochondrial green fluorescent probe (Beyotime, Shanghai, C1049) and lysosomal red fluorescent probe (Beyotime, Shanghai, C1046) were used to identify and track mitochondria and lysosomes. Following the manufacturer’s instructions, HUVECs were transfected with siRNA or plasmids for 48 h. HUVECs were labeled with probes and then treated with H2O2. Finally, the cells were observed and images were acquired using a confocal microscope (Nikon A1R, Tokyo, Japan).

### 2.20. Monocyte Adhesion Assays

HUVECs were transfected with siRNA or plasmids in a 24-well plate, and a confluent monolayer of HUVECs was formed after 48 h. Then, HUVECs were treated with TNF-α for 24 h to express adhesion factors. THP-1 cells were labeled with Calcein AM (Beyotime, Shanghai, C2012) following the manufacturer’s instructions, and then 1 × 105 THP-1 monocytes were added to a 24-well plate and incubated with HUVECs. After 30 min, the cells were washed twice with warm PBS to gently remove nonadherent cells, and then the number of adherent cells was counted using an inverted microscope.

### 2.21. Flow Cytometry

Apoptosis: The cells were collected and incubated in binding buffer, followed by staining with Annexin V-FITC and PI solution using an Annexin V-FITC Apoptosis Detection kit (Beyotime, Shanghai, C1062S). The apoptotic cells were measured by a flow cytometer, and the apoptotic rate of HUVECs at early and late apoptosis was presented as the percentage of cells with positive Annexin V-FITC staining and negative or positive PI staining.

ROS: Following the treatment, the cells were incubated with DCFH-DA for 20 min at 37 °C and then measured via flow cytometry at 488 nm excitation and 525 nm emission wavelengths.

### 2.22. Transmission Electron Microscopy Assay

HUVECs were transfected with siRNA or plasmids in a 6-well plate for 48 h. Then, HUVECs were treated with H2O2, and the cells were collected in a 1.5 mL microcentrifuge tube for each sample. After, the cell sample was fixed with 3% glutaraldehyde overnight at 4 °C and washed twice with PBS. Then, all specimens were washed in 4% (*w/v*) sucrose solution before being transferred to 1% osmium tetroxide solution. All samples were dehydrated in a series of gradients of ethanol and acetone and then embedded in epoxy resin. All samples were then cut into thin slices, stained with uranylacetate and lead citrate, and visualized with a transmission electron microscope (FEI inspect, Corvallis, OR, USA).

### 2.23. Transendothelial Resistance (TER) Measurement

HUVECs were seeded in a Transwell chamber and then measured with a transmembrane resistance meter (EVOM2, World Precision Instruments, Sarasota, FL, USA) after the cell monolayer was fully confluent. The electrodes were inserted vertically into the Transwell, with the long electrode in the lower chamber and the short electrode in the upper chamber; the value was recorded after stabilization, the measurement was repeated three times, and the average value was taken. The TER value = (measured resistance value − blank resistance value) * transwell effective membrane area.

### 2.24. Statistical Analysis

Data are presented as the mean ± standard deviation. Independent sample t-tests were performed to compare the differences using GraphPad Prism 8.0 software (GraphPad, San Diego, CA, USA). *p*-values < 0.05 indicated statistical significance.

## 3. Results

### 3.1. Oxidative-Stress-Activated MiT–TFE Transcription Factors Mediate GAS5 Expression in Sepsis

**Figure 1:**
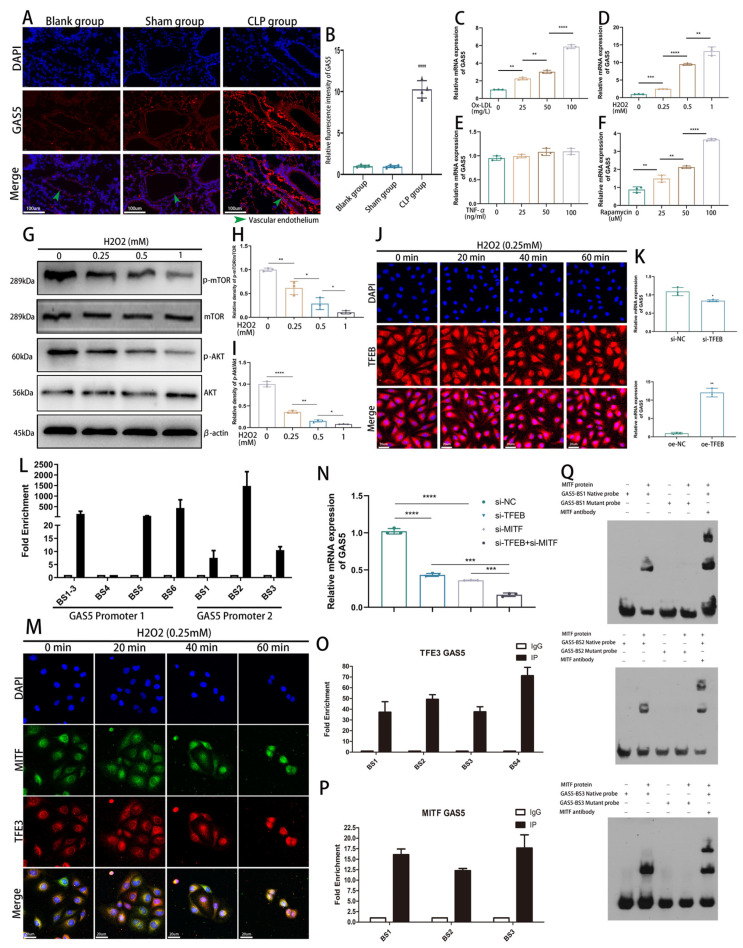
MIT–TFE family transcription factors mediate the expression of GAS5 (**A**,**B**). GAS5 expression levels in vascular endothelial cells of mice from control, sham-operated, and CLP-treated groups, measured by FISH assay 24 h post-CLP modeling, with quantification. (**C**–**F**) qPCR analysis of GAS5 expression in HUVECs after 24h exposure to Ox-LDL (**C**), H2O2 (**D**), TNF-α (**E**), and rapamycin (**F**). (**G**–**I**) Western blot assay and quantification of mTOR, p-mTOR, AKT, and p-AKT expression levels in HUVECs treated with H2O2 at different concentrations for 24 h. (**G** with quantification in **H**,**I**). (**J**) Immunofluorescence staining of TFEB distribution in H2O2-treated HUVECs. (**K**) qPCR measurement of GAS5 expression in HUVECs transfected with TFEB siRNA and TFEB overexpression plasmid for 48 h. (**L**) Endogenous binding of TFEB to promoter regions 1 and 2 of GAS5. (**M**) Immunofluorescence staining was used to track TFE3 and MITF distribution in HUVECs treated with H2O2. (**N**) GAS5 expression in HUVECs transfected with TFE3 siRNA and MITF siRNA was examined via q-PCR. (**O**,**P**) ChIP-PCR assay was used to evaluate the endogenous binding of TFE3 and MITF to the promoter region of GAS5. (**Q**) EMSA showed that MITF was directly bound to the probe containing the predicted binding motifs of GAS5. * *p* < 0.05, ** *p* < 0.01, *** *p* < 0.001, **** *p* < 0.0001.

Our studies with CLP-induced sepsis model mice revealed elevated GAS5 expression in the vascular endothelium (Figure 1A,B). Upon treating human umbilical vein endothelial cells (HUVECs) with various concentrations of oxidized low-density lipoprotein (Ox-LDL), we found a positive correlation between GAS5 expression and Ox-LDL concentration (Figure 1C). Considering that the predominant detrimental effects of Ox-LDL encompass oxidative stress and inflammation, we simulated these effects in HUVECs using H2O2 and TNFα. Interestingly, our results demonstrated that only H2O2 was capable of inducing GAS5 expression (Figure 1D,E). Moreover, the autophagy agonist rapamycin also upregulated GAS5 expression in HUVECs (Figure 1F).

We further confirmed that H2O2 activated the autophagy response in ECs (Figure S1A–F). Our findings suggested a potential link between sepsis-induced GAS5 expression and the protective autophagy mechanism triggered by oxidative stress. As a redox sensor, the PI3K–Akt–mTOR signaling pathway was significantly inhibited after H2O2 treatment, suggesting mTORC1 function suppression (Figure 1G–I). Next, we silenced Raptor, a key adaptor protein in the mTORC1 complex, to inhibit mTORC1 function and observed elevated GAS5 expression (Figure S1G). Hence, we hypothesized that TFEB, a downstream transcription factor of mTORC1, mediated GAS5 transcription.

Following H2O2 treatment in ECs, TFEB translocated to the nucleus over time (Figure 1J). Subsequently, upon TFEB knockdown, GAS5 expression levels declined by ~20%; however, TFEB overexpression led to a significant increase in GAS5 expression (Figure 1K). We identified two main promoter regions (promoter region 1 and region 2) for GAS5 transcripts with distinct transcription start sites (TSSs) (Figure S1H). ChIP-PCR further confirmed that TFEB bound to both promoters of GAS5 and participated in GAS5 transcription (Figure 1L).

We speculated that the limited impact of TFEB knockdown on GAS5 expression might be attributed to the potential involvement of other MiT–TFE family members, such as MITF and TFE3, in GAS5 transcription. Our assessment of MITF and TFE3 cellular localization under oxidative stress indicated their rapid nuclear translocation for transcriptional activity (Figure 1M). Knockdown of MITF or TFE3 can cause a significant reduction in the expression level of GAS5 (Figure 1N). Binding sites of TFE3 and MITF in the GAS5 promoter region (Figure S1I), retrieved from the JASPAR database, were validated by ChIP-PCR results, indicating that both transcription factors have binding sites on the GAS5 promoter (Figure 1O,P). Further support derived from EMSA, which confirmed a direct interaction between MITF and the transcription-factor-binding site in the GAS5 promoter region (Figure 1Q).

It is noteworthy that, contrary to the widely held belief of a negative correlation between p62 levels and autophagy levels, p62 levels actually exhibited a biphasic trend, decreasing initially and then increasing as ROS-induced autophagy levels escalated (Figure S1C). This phenomenon might suggest that oxidative-stress-induced autophagy has a mechanism to compensate for the consumption of adaptor proteins.

### 3.2. GAS5 Exhibits Antioxidant and Anti-Inflammatory Roles in Sepsis

**Figure 2:**
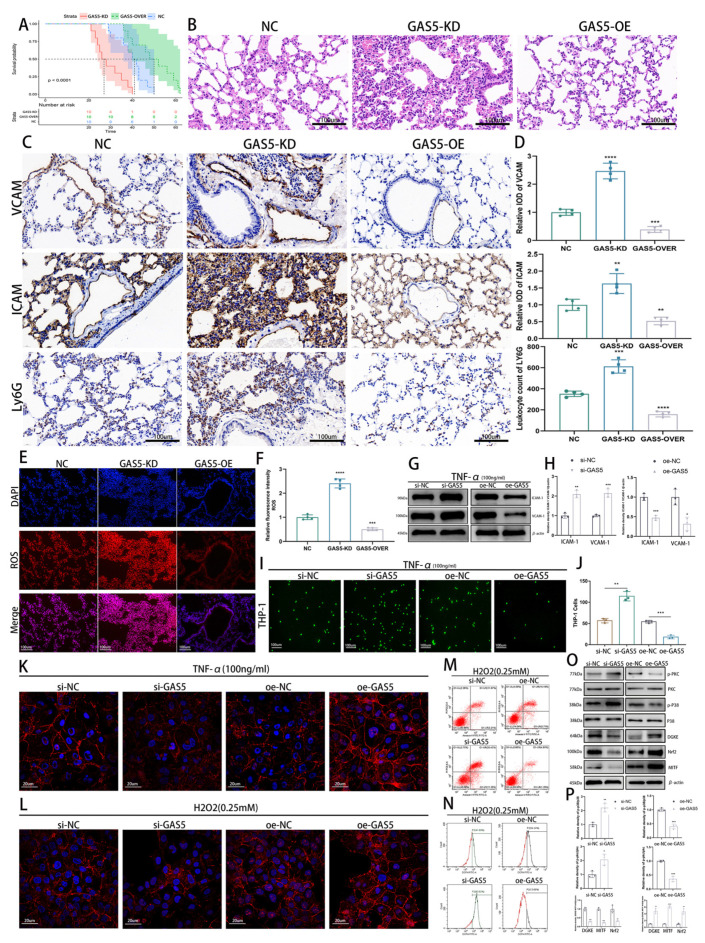
Antioxidant and anti-inflammatory effects of GAS5 in sepsis. (**A**) Survival analysis of mice in NC, GAS5-KD, and GAS5-OVER groups after CLP modeling (n = 10 per group), with plotted survival curves. (**B**) Hematoxylin and eosin staining of lung tissue from NC, GAS5-KD, and GAS5-OVER groups 24 h post-CLP surgery. (**C**,**D**) Immunohistochemical staining and quantification of ICAM-1, VCAM-1, and Ly6G in lung tissue of NC, GAS5-KD, and GAS5-OVER groups 24 h post-CLP surgery. (**E**,**F**) ROS levels in lung tissue from different groups 24 h after CLP surgery, with quantification. (**G**,**H**) Western blot analysis and quantification of ICAM-1 and VCAM-1 expression in HUVECs after 24 h TNF-α treatment. (**I**,**J**) Monocyte adhesion assay assessing HUVEC adhesion to THP-1 monocytes following 24 h TNF-α treatment, with quantification. (**K**) Effects of GAS5 on endothelial cell intercellular junctions after 24 h TNF-α treatment. (**L**) Effects of GAS5 on endothelial cell intercellular junctions after 24 h H2O2 treatment. (**M**) Flow cytometry assessment of apoptosis in HUVECs under oxidative stress with GAS5 modulation. (**N**) Flow cytometry evaluation of ROS levels in HUVECs under oxidative stress with GAS5 modulation. (**O**,**P**) Western blot analysis of the expression levels of MITF, DGKE, Nrf2, p38, p-p38, PKC, and p-PKC in HUVECs transfected with GAS5 siRNA and the GAS5 overexpression plasmid (**O** with quantification in **P**). * *p* < 0.05, ** *p* < 0.01, *** *p* < 0.001, **** *p* < 0.0001.

C57 mice infected with AAV vectors targeting vascular endothelium exhibited effective expression in lung tissue (Figure S2A). GAS5 overexpression (GAS5-OVER) extended survival in septic injury compared to the control, while knockdown shortened it. Post-CLP surgery, 90% of GAS5-knockdown mice succumbed within 40 h, compared to 40% of wild-type (WT) mice. By the 50 h mark, 90% of WT mice had died, whereas only 50% of GAS5-OVER mice met the same fate, with some surviving beyond 60 h (Figure 2A).

HE staining revealed that GAS5 mitigated sepsis-induced vascular leakage, reduced lung injury, and preserved lung tissue structural integrity (Figure 2B). GAS5 overexpression reduced adhesion molecule expression, including ICAM and VCAM, and suppressed inflammatory cell infiltration in lung tissue (Figure 2C,D). Moreover, GAS5 knockdown in early sepsis caused a surge in lung ROS levels, whereas GAS5 overexpression reduced ROS levels (Figure 2E,F).

To simulate sepsis-induced inflammation in vitro, ECs were treated with TNFα. The leukocyte adhesion assay showed that GAS5 overexpression suppressed ICAM and VCAM expression, reducing EC–leukocyte adhesion (Figure 2G–J). The expression of GAS5 alleviated the inflammatory factor- or ROS-induced remodeling of intercellular junctions, reducing sepsis-induced pulmonary vascular leakage (Figure 2K,L). Flow cytometry results showed that GAS5 overexpression inhibited oxidative stress-induced apoptosis and reduced intracellular ROS levels (Figure 2M,N). Upon knockdown of GAS5, we observed an inhibition of lysosomal signaling and activation of inflammation-related mitogen-activated protein kinase (MAPK) signaling, as demonstrated by high-throughput sequencing (Figure S2B). Subsequently, we generated heatmaps of the main differentially expressed genes related to these signaling pathways (Figure S2C) and verified their authenticity with Western blotting (Figure 2O,P).

### 3.3. Existence of a MITF–GAS5–miR-23 Positive Feedback Loop in Endothelial Cells

**Figure 3:**
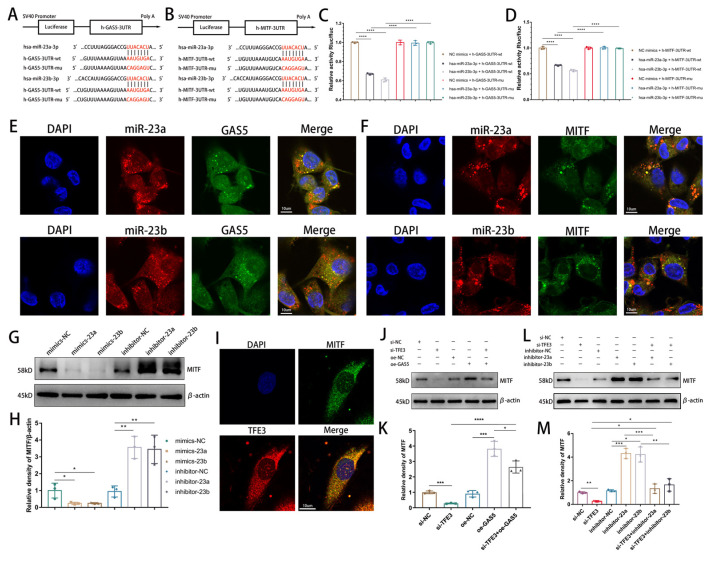
Verification of the MITF–GAS5–mir23 positive feedback loop in vascular endothelial cells. (**A**) The predicted binding site of miR-23a and miR-23b on GAS5. (**B**) The predicted binding site of miR-23a and miR-23b on MITF. (**C**) The luciferase reporter gene assay confirmed the direct interaction of miR-23a and miR-23b with GAS5. (**D**) The luciferase reporter gene assay confirmed the direct interaction of miR-23a/b with MITF. (**E**) Intracellular colocalization of GAS5 and miR-23 detected through a FISH assay. (**F**) Intracellular colocalization of MITF and miR-23 detected by FISH assay. (**G**,**H**) Western blot analysis of the expression levels of MITF in HUVECs transfected with miR-23 mimics and inhibitors. (**I**) Fluorescent antibodies were used to label TFE3 and MITF to observe their cellular colocalization. (**J**,**K**) The expression of MITF was analyzed by Western blot analysis after transfection of HUVECs with TFE3 siRNA and the GAS5-overexpression plasmid (**J** with quantification in **K**). (**L**,**M**) HUVECs were transfected with si-TFE3, miR-23a/b inhibitor, and GAS5-overexpression plasmid, and the expression of MITF was analyzed via Western blot analysis (**L** with quantification in **M**). * *p* < 0.05, ** *p* < 0.01, *** *p* < 0.001, **** *p* < 0.0001.

Upon examining differentially expressed microRNAs (miRNAs), we noted a significant upregulation of the miR-23 family (miR-23a, miR-23b) following the knockdown of GAS5 (Figure S3A). As per bioinformatics predictions, not only can miR-23 be sponged by GAS5 (Figure 3A), but it also has a binding site in the 3′ untranslated region (3′UTR) of MITF (Figure 3B). Subsequently, we used dual luciferase reporter gene assays to confirm the interaction between GAS5 and miR-23 (Figure 3C) and further confirmed that miR-23 directly binds to the 3′ UTR of MITF (Figure 3D). Next, we labeled miR-23a, miR-23b, GAS5, and MITF with specific FISH probes. FISH assay results revealed colocalization between GAS5 and miR-23, as well as miR-23 and MITF (Figure 3E,F). Western blot showed that miR-23 mimics could inhibit the expression of MITF in HUVECs, and the miR-23 inhibitor could augment the expression of MITF (Figure 3G,H). To conclude, we found that GAS5, transcribed by MITF and TFE3, shields MITF mRNA from miR-23-mediated degradation, potentially establishing a positive feedback loop to enhance MITF expression.

To further verify the existence of the MITF/TFE3–GAS5–miR-23 positive feedback loop, we labeled MITF and TFE3 with fluorescent antibodies, confirming their ability to form heterodimers (Figure 3I). If this loop exists, TFE3 knockdown would compromise dimer functionality, subsequently blocking the loop and leading to a reduction in MITF expression. Before evaluating this hypothesis, we needed to demonstrate that GAS5 and miR-23 possess an upstream–downstream relationship in regulating MITF. We confirmed that miR-23 mimics reversed the elevation of MITF expression levels induced by GAS5 overexpression (Figure S3B,C) and further validated that both GAS5 and miR-23 inhibitors could counteract the decrease in MITF expression levels resulting from TFE3 knockdown (Figure 3J–M).

### 3.4. GAS5 Exerts Anti-Inflammatory Effects through the GAS5–miR-23–DGKE Axis

**Figure 4:**
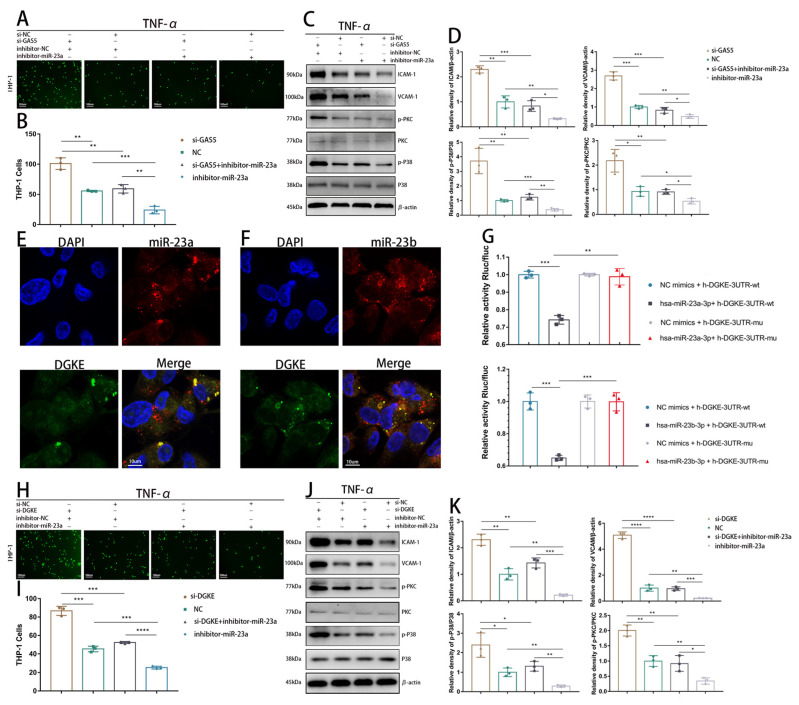
The GAS5–miR-23–DGKE axis is responsible for the anti-inflammatory effects of GAS5 (**A**–**D**) HUVECs in the control group, GAS5 knockdown group, miR-23a inhibitor group, and GAS5-KD+miR-23a inhibitor group were treated with TNF-α. (**A**,**B**) A monocyte adhesion test was used to evaluate the adhesion ability of HUVECs to THP-1 monocytes under inflammation (**A** with quantification in **B**). (**C**,**D**) Western blot analysis was used to evaluate the expression levels of p38, p-p38, PKC, p-PKC, ICAM-1 and VCAM-1 in HUVECs (**C** with quantification in **D**). (**E**) Intracellular colocalization of DGKE and miR-23a detected by FISH assay. (**F**) Intracellular colocalization of DGKE and miR-23b detected through a FISH assay. (**G**) The luciferase reporter gene assay confirmed the direct interaction of miR-23a/b with DGKE. (**H**–**K**) HUVECs in the control group, DGKE knockdown group, miR-23a inhibitor group, and DGKE-KD+miR-23a inhibitor group were treated with TNF-α. (**H**,**I**) A monocyte adhesion test was used to evaluate the adhesion ability of HUVECs to THP-1 monocytes under inflammation (**H** with quantification in **I**). (**J**,**K**) Western blot analysis was used to evaluate the expression levels of p38, p-p38, PKC, p-PKC, ICAM-1, and VCAM-1 in HUVECs (**J** with quantification in **K**). * *p* < 0.05, ** *p* < 0.01, *** *p* < 0.001, **** *p* < 0.0001.

Functional experiments were conducted to confirm that GAS5, which can regulate the anti-inflammatory gene DGKE, achieves its anti-inflammatory effect by alleviating miR-23’s inhibitory impact on DGKE. Monocyte adhesion assays revealed that miR-23 inhibitor overexpression reduced EC adherence to monocytes, and reversed the increase in monocyte adhesion caused by GAS5 knockdown (Figure 4A,B and Figure S4A,B). Additionally, Western blot analysis showed that the overexpression of the miR-23 inhibitor reduced the expression of adhesion factors such as ICAM and VCAM by inhibiting the P38 MAPK pathway. Additionally, the overexpression of the miR-23 inhibitor also inhibited the PKC pathway and reversed the overactivation of PKC and P38 caused by GAS5 knockdown, partly explaining the antioxidant effect of GAS5 (Figure 4C,D and Figure S4C,D).

Bioinformatics analysis showed miR-23 has binding sites in the 3’UTR of DGKE mRNA (Figure S4E). FISH probes labeled with miR-23a, miR-23b, and DGKE showed that GAS5 and miR-23 co-localized (Figure 4E,F). Western blot analysis showed that miR-23 mimics reduced DGKE expression while the miR-23 inhibitor increased DGKE expression levels (Figure S4F,G). We utilized dual luciferase reporter gene experiments to show that miR-23 directly interacted with the 3’UTR of DGKE mRNA (Figure 4G). Subsequent monocyte adhesion experiments demonstrated that silencing DGKE expression exacerbated monocyte adherence during inflammation and that these effects were reversed by the overexpression of the miR-23 inhibitor (Figure 4H,I and Figure S4H,I). Western blot assays revealed that the overexpression of the miR-23 inhibitor reversed the activation of the P38 and PKC pathways caused by DGKE knockdown (Figure 4J,K and Figure S4J,K).

### 3.5. GAS5 Exerts Antioxidant and Mitochondrial Quality Control (MQC) Effects via a Mitophagy-Independent Mechanism

**Figure 5:**
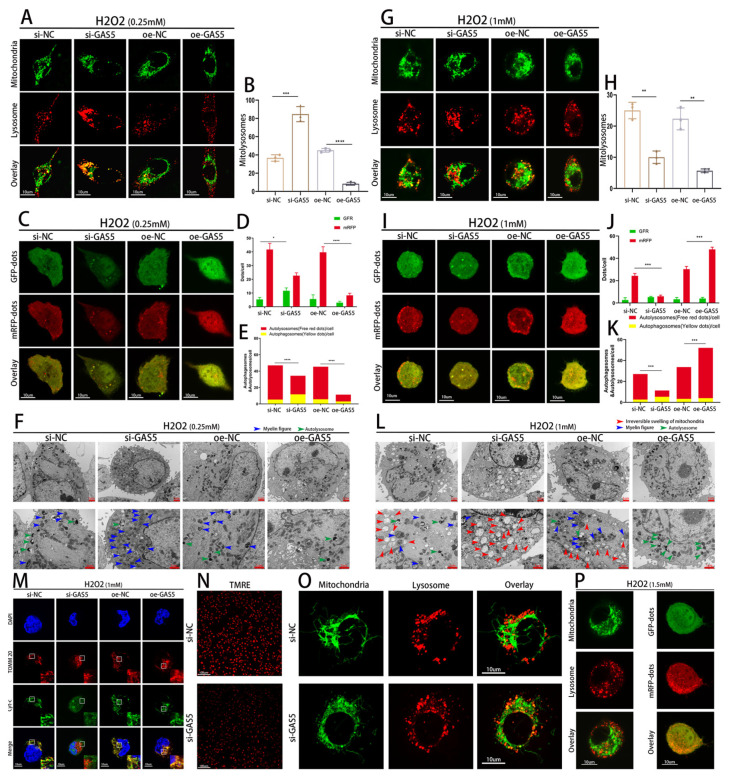
Mitophagy-independent mechanism enables GAS5 to exert antioxidant and mitochondrial quality control (MQC) effects. (**A**,**B**,**G**,**H**) Green and red fluorescent probes label mitochondria and lysosomes, respectively, to observe mitophagy levels after 1 h of H2O2 treatment, with quantification. (**C**–**E**,I–**K**) HUVECs transfected with mRFP–GFP–LC3 adenovirus to observe autophagic flux levels after 1 h of H2O2 treatment, with quantification. (**F**–**L**) Transmission electron microscopy was used to assess the type of autophagy and the degree of mitochondrial damage. (**M**) Fluorescent antibodies were used to label TOM20 and cyt C to assess the extent of cyt C diffusion from mitochondria to the cytoplasm after 1 h of H2O2 treatment. (**N**) Labeling of the TMRE probe was used to evaluate mitochondrial membrane potential (Δψm). (**O**) Mitochondria and lysosomes were labeled with green and red fluorescent probes, respectively, to assess the degree of mitophagy following GAS5 knockdown in the absence of exogenous ROS. (**P**) The concentration of H202 was increased to assess the level of mitophagy and total autophagy levels in GAS5-overexpressing HUVECs. * *p* < 0.05, ** *p* < 0.01, *** *p* < 0.001, **** *p* < 0.0001.

We investigated whether GAS5 is involved in MQC through enhancing p62-mediated mitophagy. Contrary to our expectations, under OS, GAS5 knockdown increased the levels of mitophagy, while GAS5 overexpression inhibited it (Figure 5A,B). To assess the effect of GAS5 on the overall level of autophagy, ECs were infected with the adenovirus mRFP–GFP–LC3. Under oxidative stress, the GAS5 knockdown group exhibited a higher number of autophagosomes, while the control group displayed more autolysosomes. Concurrently, the GAS5 knockdown group had a larger autophagosome volume, indicative of increased organelle autophagic degradation, which aligned with elevated mitophagy. Conversely, ECs overexpressing GAS5 demonstrated no significant autophagy activity under oxidative stress (Figure 5C–E). Transmission electron microscopy can spot oxidative-damage-related alterations of the mitochondrial membrane, known as myelin-like figures. The GAS5 knockdown group showed a notable increase in intracellular myelin-like figures compared to the control group, while GAS5 overexpression hindered their formation (Figure 5F).

Interestingly, we discovered that raising H2O2 concentration switched the impact of GAS5 knockdown on mitophagy from facilitative to inhibitory. Under severe oxidative stress, GAS5 knockdown led to a considerable decrease in mitophagy levels, while GAS5 overexpression continued to impede mitophagy occurrence (Figure 5G,H). Besides mitophagy, under severe oxidative stress, GAS5 knockdown also resulted in reduced overall autophagy levels, suggesting autophagy function impairment. Compared to the control group, the GAS5 overexpression group exhibited a higher count of autolysosomes with a smaller volume (Figure 5I–K). Subsequently, transmission electron microscopy revealed that under this level of oxidative stress, GAS5 knockdown worsened irreversible mitochondrial swelling, with no mitophagy-mediated MQC observed. Compared to the control group ECs, GAS5-overexpressing ECs did not exhibit mitochondrial myelin-like changes or irreversible swelling (Figure 5L).

We subsequently demonstrated that under severe oxidative stress, cytochrome c (cyt C) and the mitochondrial protein TOMM20 were largely colocalized in GAS5-overexpressing ECs, as shown by immunofluorescence, whereas loss of GAS5 resulted in abundant cyt C leakage from mitochondria, which can lead to EC apoptosis (Figure 5M). We further found that GAS5 knockdown resulted in a marked decrease in mitochondrial membrane potential (Δψm) (Figure 5N), manifesting as mitophagy activity even in the absence of H2O2 induction (Figure 5O), which means that the observed enhanced mitophagy is the result of oxidative damage rather than that of an antioxidant mechanism. As oxidative stress intensified, the discrepancy between mitophagy and overall autophagy intensity in GAS5-overexpressing ECs persisted (Figure 5P), suggesting that mitophagy is not the prioritized autophagy type to combat oxidative stress.

### 3.6. GAS5 Integrates the Nrf2 Antioxidant Pathway and Autophagy System through the GAS5–MITF Axis

**Figure 6:**
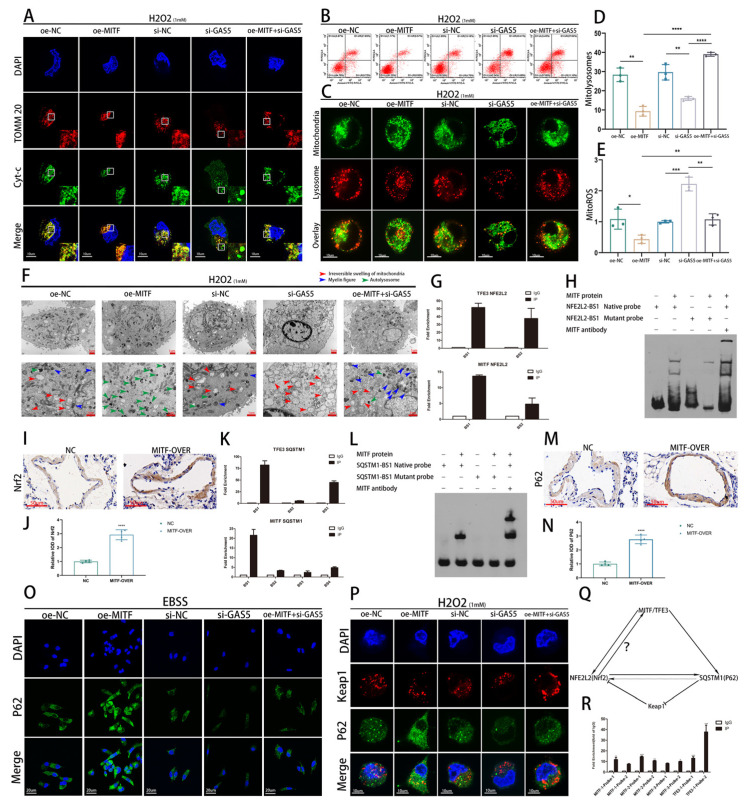
The GAS5–MITF axis integrates the Nrf2 antioxidant pathway and autophagy system. (**A**–**F**) HUVECs in the control group, MITF overexpression group, GAS5 knockdown group, and GAS5–KD+MITF-OVER group were treated with H2O2 for 1 h. (**A**) Fluorescent antibodies were used to label TOMM20 and cyt C to assess the extent of cyt C diffusion from mitochondria to the cytoplasm after 1 h of H2O2 treatment. (**B**) Flow cytometry was used to assess apoptosis levels after 1 h of H2O2 treatment. (**C**,**D**) Green and red fluorescent probes were used to label mitochondria and lysosomes, respectively, to evaluate the level of mitophagy after 1 h of H2O2 treatment (**C** with quantification in **D**). (**E**) MitoSOX™ Red Mitochondrial Superoxide Indicator was used to evaluate the levels of mitochondrial ROS after 1 h of H2O2 treatment. (**F**) Transmission electron microscopy was used to assess the type of autophagy and the degree of mitochondrial damage. (**G**) ChIP-PCR assay was used to evaluate the endogenous binding of TFE3 and MITF to the promoter of the NFE2L2 gene. (**H**) The EMSA results showed that MITF was directly bound to the probe containing the predicted binding motifs of NFE2L2. (**I**,**J**) Immunohistochemical staining of Nrf2 in the NC group and MITF-overexpressing groups (**I** with quantification in **J**). (**K**) A ChIP-PCR assay was used to detect the endogenous binding of TFE3 and MITF to the promoter of the SQSTM1 gene. (**L**) The EMSA results showed that MITF was directly bound to the probe containing the predicted binding motifs of SQSTM1. (**M**,**N**) Immunohistochemical staining of p62 in the NC group and MITF-overexpressing groups (**M** with quantification in **N**). (**O**) Immunofluorescence was used to evaluate the fluorescence intensity and intracellular distribution of p62. (**P**) Fluorescent antibodies were used to label Keap1 and p62 and observe their cellular colocalization. (**Q**) Schematic diagram of the regulation of MITF, Nrf2, and P62 (**R**) A ChIP-PCR assay was used to detect the endogenous binding of Nrf2 to the promoters of TFE3 and MITF. * *p* < 0.05, ** *p* < 0.01, *** *p* < 0.001, **** *p* < 0.0001.

We found that GAS5 mainly relies on MITF to perform its antioxidant and autophagy-regulation functions. Enhanced mitochondrial cyt C leakage under oxidative stress was observed upon GAS5 silencing, while this effect was mitigated by MITF overexpression (Figure 6A). Flow cytometry analysis of apoptosis reinforced that MITF counteracts cell apoptosis induced by GAS5 knockdown (Figure 6B). Although MITF overexpression did not directly enhance mitophagy levels, it restored the impaired mitophagy function resulting from GAS5 silencing (Figure 6C,D). MITF overexpression diminished mitochondria-derived ROS levels and averted ROS-induced alterations in the mitochondrial membrane potential (Δψm) (Figure 6E and Figure S5A). Transmission electron microscopy revealed that MITF overexpression hindered irreversible mitochondrial swelling due to GAS5 knockdown (Figure 6F).

In addition to serving as an autophagy-related transcription factor, MITF exhibits an antioxidant mechanism that works in concert with autophagy regulation. Given the MITF–GAS5–miR-23 loop, differentially expressed genes following GAS5 knockdown could be MITF targets. As GAS5 knockdown reduced the antioxidant transcription factor Nrf2 expression, we hypothesize that MITF may also participate in NFE2L2 (Nrf2) transcription. JASPAR database predictions indicate that both MITF and TFE3 possess transcription factor binding sites in the NFE2L2 promoter region (Figure S5B). Using ChIP-PCR and EMSA, we verified these binding sites in the NFE2L2 promoter region (Figure 6G,H). Immunohistochemical staining showed elevated Nrf2 expression levels after AAV-mediated MITF overexpression (Figure 6I,J).

The downregulation of SQSTM1 (p62) expression following GAS5 knockdown is noteworthy. JASPAR database analysis revealed that both TFE3 and MITF have binding sites in the SQSTM1 promoter region (Figure S5C). ChIP-PCR confirmed TFE3 and MITF binding to the SQSTM1 promoter region (Figure 6K). Using EMSA, we further verified high-affinity MITF transcription factor binding sites in the promoter region based on ChIP-PCR results and confirmed direct binding (Figure 6L). Immunohistochemical staining demonstrated increased p62 expression levels after MITF overexpression (Figure 6M,N).

Based on current experimental data, the transcription of p62 via the GAS5–MITF axis does not directly augment mitophagy levels. To assess the p62 reserve, we subjected the ECs to Earle’s balanced salt solution (EBSS), an effective inducer of autophagy. The subsequent results unveiled a significant surge in both p62 fluorescence intensity and the number of p62-mediated autophagosomes in cells overexpressing MITF (Figure 6O). Although the MITF–p62 pathway may not generally amplify mitophagy, it might, under oxidative stress conditions, potentially modulate the p62–Keap1–Nrf2 pathway, leading to an increase in Nrf2 expression at the post-transcriptional level. Fluorescent antibodies were used to label p62 and Keap1. Under severe oxidative stress, GAS5-deficient ECs did not effectively mediate the degradation of Keap1. Following MITF overexpression, p62-mediated autophagy activity adequately mediated Keap1 degradation (Figure 6P). Previous studies have shown that Nrf2 can mediate the transcription of SQSTM1 (P62). Our experimental findings suggest the existence of a triangular regulatory system among Nrf2, MITF, and p62, wherein these components appear to exert reciprocal regulatory effects on one another. Given that MITF/TFE3 has been shown to mediate Nrf2 transcription, it can be inferred from the principle of reciprocal regulation that Nrf2 might also possess the capability to transcribe MITF/TFE3 (Figure 6Q). Subsequently, we employed ChIP-PCR to further ascertain the specific binding sites of Nrf2 within the promoter regions of MITF and TFE3 (Figure S5D). Our results confirmed the direct binding interaction, thereby providing robust evidence for our hypothesis (Figure 6R).

### 3.7. The MITF–GAS5–miR-23 Positive Feedback Loop Protects ECs during Sepsis

**Figure 7:**
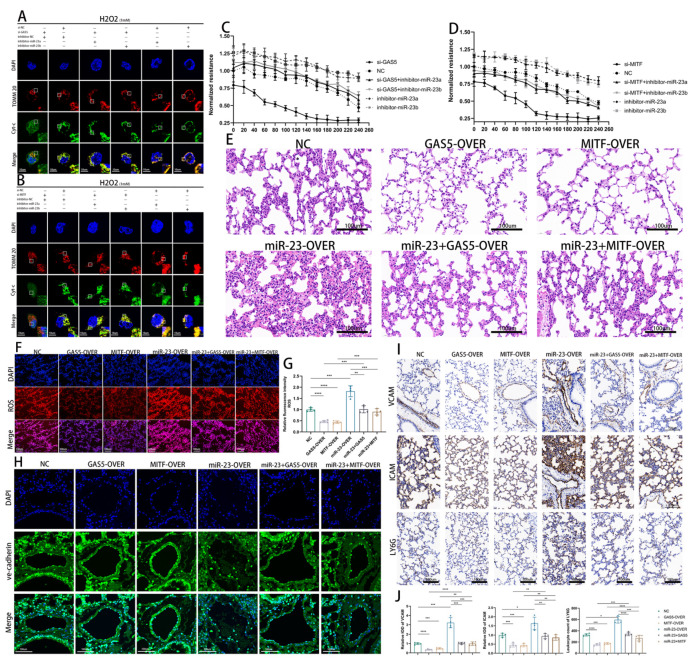
In vivo validation of the protective effect of the MITF–GAS5–miR-23 positive feedback loop. (**A**–**H**) HUVECs in the control group, GAS5 knockdown group, miR-23a/b inhibitor group, GAS5-KD+miR-23a/b-inhibitor group, MITF knockdown group, and MITF–KD+miR-23a/b-inhibitor group were treated with H2O2. (**A**,**B**) Fluorescent antibodies labeled TOM20 and cyt C, respectively, to assess the extent of cyt C diffusion from mitochondria to the cytoplasm. (**C**,**D**) The trend of normalized transendothelial electrical resistance (TER) of HUVECs over time after H2O2 treatment. (**E**–**I**) A CLP sepsis model was established in mice in the NC group, GAS5-OVER group, MITF-OVER group, miR-23 group, miR-23+GAS5-OVER group, and miR-23+MITF-OVER group. (**E**) Lung tissue was isolated and stained with hematoxylin and eosin after CLP surgery. (**F**,**G**) The level of ROS in the lung tissue of different groups of mice after the CLP operation (**F** with quantification in **G**). (**H**) Immunofluorescence staining of VE-cadherin was used to assess vascular barrier function and the results showed continuity of intercellular junctions. (**I**,**J**) Immunohistochemical staining of ICAM-1, VCAM-1, and Ly6G in the NC group, GAS5-OE group, MITF-OE group, miR-23 group, miR-23+GAS5-OVER group, and miR-23+MITF-OVER group after CLP surgery (**I** with quantification in **J**). * *p* < 0.05, ** *p* < 0.01, *** *p* < 0.001, **** *p* < 0.0001.

To confirm the role of the MITF–GAS5–miR-23 loop in sepsis protection, we conducted follow-up experiments. Under oxidative stress, we observed that knockdown of GAS5 or MITF led to a pronounced leakage of mitochondrial cyt C and its widespread distribution in the cytoplasm, thereby inducing apoptosis. However, miR-23 (miR-23a and miR-23b) inhibitor expression maintained TOMM20 and cyt C localization consistency and reversed the cyt C leakage caused by GAS5 or MITF knockdown (Figure 7A,B). Likewise, the levels of mitochondrial ROS exhibited a GAS5–miR-23–MITF axis-dependent correlation, and the increase in mitochondrial ROS induced by silencing GAS5 or MITF could be reversed by miR-23 inhibitor expression (Figure S6A–D). The transmembrane resistance assay results showed a decline in the overall transmembrane resistance of vascular ECs after H2O2 treatment. Compared to the control group, ECs with miR-23 inhibitor overexpression exhibited the better maintenance of transmembrane resistance and reversed the loss of transmembrane resistance caused by GAS5 or MITF knockdown after 4 h of H2O2 treatment (Figure 7C,D and Figure S6E,F).

In vivo, AAV vectors were used to target miR-23 (miR-23a and miR-23b), GAS5, and MITF expression in mouse vascular endothelium. HE staining results indicated that, compared to the control group, miR-23 overexpression exacerbated sepsis-induced lung tissue damage, manifested as more severe leakage and tissue structural damage. Conversely, GAS5 and MITF overexpression both mitigated miR-23-mediated septic lung injury (Figure 7E). The evaluation of ROS levels in lung tissue also showed that lung tissue with a high expression of MITF or GAS5 showed lower ROS levels under sepsis, while lung tissue overexpressing miR-23 displayed noticeably higher ROS levels (Figure 7F,G). ROS and inflammation can lead to alterations in vascular permeability. To evaluate the impact of the MITF–GAS5–miR-23 loop on vascular permeability during sepsis, fluorescent antibodies labeled Ve-cadherin. The overexpression of miR-23 impaired the vascular barrier function, causing a marked Ve-cadherin continuity impairment. In contrast, GAS5 or MITF overexpression stabilized ECs junctions and alleviated miR-23-induced endothelial barrier dysfunction (Figure 7H). Finally, we identified inflammation factors and inflammatory cell markers, and the results demonstrated that the MITF–GAS5–miR-23 loop regulates the anti-inflammatory reaction to sepsis as anticipated (Figure 7I,J).

## 4. Discussion

Based on the known biological mechanisms of Ox-LDL, H2O2, and rapamycin, we hypothesized that autophagy-associated transcription factors activated under oxidative stress contribute to GAS5 transcription. While existing knowledge highlights the role of the redox–sensor mTORC1 complex in regulating TFEB—a member of the MiT-TFE family—in response to ROS stimulation [36], our investigations suggested that TFEB silencing had minimal influence on GAS5 expression in endothelial cells (ECs). Analysis from sequencing data showed that expression levels of TFEB and TFEC were comparatively lower in ECs than MITF and TFE3, suggesting that the latter were the primary contributors to GAS5 transcription.

During sepsis, mitochondria, as the main source of intracellular ROS, are particularly vulnerable to active molecules released by activated immune cells [39]. We observed that GAS5 upregulated autophagy adapter protein and ECs with enhanced GAS5 expression typically maintain healthier mitochondrial populations. This sparked a pertinent inquiry: Does GAS5 regulate mitochondrial quality control (MQC) via mitophagy? However, our subsequent data seemed to counter this hypothesis, indicating that GAS5 predominantly suppressed mitophagy. The manifestation of mitophagy after GAS5 silencing seemed to be tied to the severity of oxidative stress. Under mildly oxidative conditions, GAS5 knockdown prompted mitochondria to transform into myelin-like figures, coupled with amplified mitophagy, suggesting that GAS5 silencing exacerbates oxidative damage but leaves the mitophagy mechanism intact. As oxidative stress escalated, the absence of GAS5 triggered irreversible mitochondrial swelling, alongside impaired mitophagy, culminating in cell death. In contrast, ECs that overexpressed GAS5 neither display mitochondrial swelling nor myelin-like figures, with only typical autolysosomes observed. We proposed that GAS5’s function represented a synergistic effect arising from MITF’s transcription of multiple genes. Notably, mitophagy was not the primary choice, as MITF additionally mediated Nrf2 transcription, which in turn inhibited mitochondrial oxidative damage via the MITF–Nrf2–ARE pathway. Concurrently, the normal functioning of mitophagy necessitated the antioxidant mechanism, providing a relatively stable intracellular environment.

In the context of sepsis-induced oxidative damage, what is the functional significance of the MITF–GAS5–miR-23 loop-activated p62 expression? Current research has revealed a transient increase in autophagy during sepsis, followed by a long-term decline in autophagic flux, ultimately resulting in organ dysfunction [40,41,42]. To date, the mechanisms leading to the decline in autophagic flux in sepsis remain to be studied. One potential explanation is that overactive autophagy in the initial stage depletes autophagy-related proteins, rendering them unable to be effectively replenished later [43]. Our experimental findings indicated that under oxidative stress, the MITF–GAS5–miR-23 loop persistently elevated levels of autophagy-related proteins, while the high expression of p62 did not yield a transient increase in autophagy or mitophagy levels. On the contrary, autophagy levels were dynamically regulated in response to the intensity of oxidative stress. The high expression of MITF or GAS5 correlated with relatively lower autophagy intensity, attributable to the antioxidant effect of MITF-transcribed Nrf2, which in turn contributes to the maintenance of autophagic flux during sepsis.

**Figure 8:**
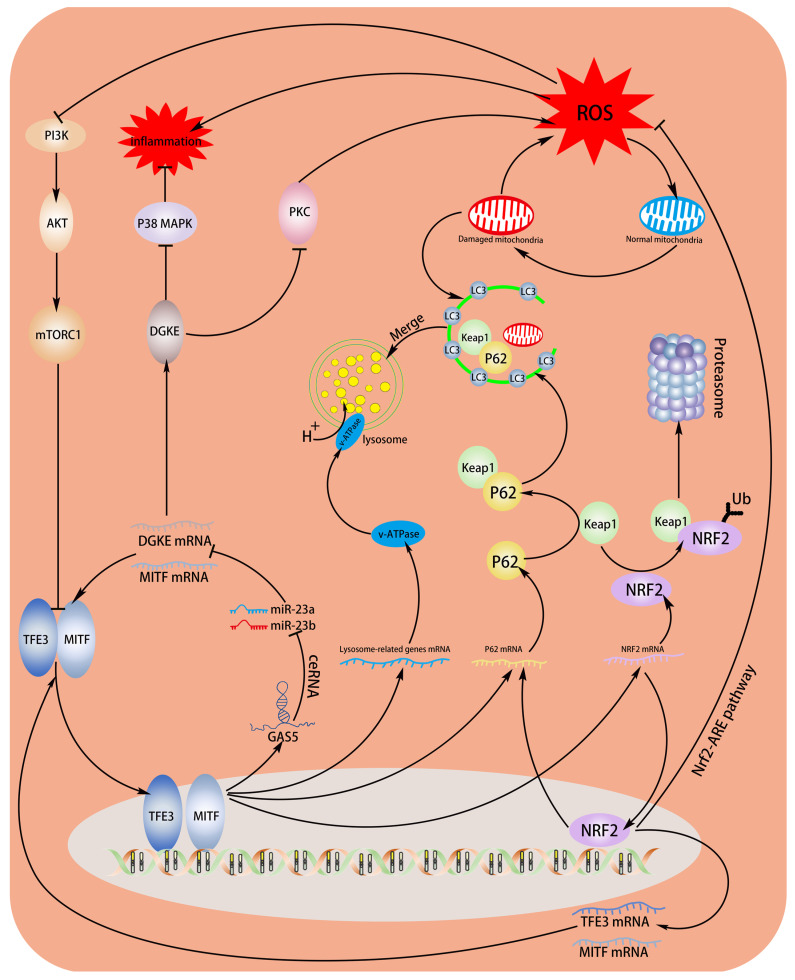
A model for the anti-inflammatory and anti-oxidative mechanisms of the MITF–GAS5–miR-23 loop in sepsis vascular endothelium. As shown in this figure, after endothelial cells are stimulated by ROS, the pi3k–Akt–mTOR signaling pathway is inhibited, causing the MiT–TFE transcription factor to enter the nucleus, participate in the transcription of GAS5, and form a positive feedback loop. At the same time, the transcription of Nrf2 and P62 is also activated, and autophagy behavior is dynamically regulated according to the degree of oxidative stress. In addition, a mutually fostering transcription mechanism exists between Nrf2 and MiT–TFE transcription factors, amplifying the effect of the MITF–GAS5–miR-23 loop.

Despite the GAS5 inhibition of mitophagy, numerous autolysosomes were still present in the cytoplasm. This suggests the possibility of other types of autophagy that may exert antioxidant effects, potentially superior to mitophagy. As previously reported, p62 can bind to Keap1, inhibiting Keap1-mediated polyubiquitinylation, and the subsequent proteasomal degradation of Nrf2 [16]. Our observations further revealed that p62-mediated autophagy resulted in more pronounced Keap1 degradation in GAS5-high-expressing ECs, explaining the asynchronous levels of mitophagy and autophagy and showed that the MITF–GAS5–miR-23 loop enhanced Nrf2 expression via P62-mediated post-transcriptional mechanisms. Besides MITF, Nrf2 also mediated the transcription of p62, thus forming a positive feedback loop of p62–Keap1–Nrf2 [17]. We further propose that MITF, p62, and Nrf2 formed a triangular regulatory system (hereinafter referred to as the “MITF–Nrf2–p62 triangle”). Upon confirming the transcriptional effects of Nrf2 on MITF and TFE3, we observed that the MITF–Nrf2–p62 triangle components exhibited a pairwise mutual regulation pattern, thus enriching the regulatory theory of the MITF–Nrf2–p62 triangle. In summary, the protective effect of GAS5 in sepsis is mainly the result of the synergistic effect of the target gene transcribed by MITF. The triangular regulatory system comprising MITF, Nrf2, and p62 integrates antioxidant and autophagy mechanisms, increasing autophagy adaptor protein reserves while preventing transient autophagy surges caused by oxidative stress, thus contributing to the maintenance of autophagic flux in sepsis (Figure 8 A model for the anti-inflammatory and anti-oxidative mechanisms of the MITF–GAS5–miR-23 loop in sepsis vascular endothelium). The selective regulation of autophagy by GAS5 under oxidative stress has developed the theory of sepsis-related autophagy regulation, and the biological role of GAS5 in anti-oxidation and anti-inflammation also suggests that GAS5 is expected to become a new target for the treatment of sepsis.

## Data Availability

The authors declare that all data supporting the findings of this study are available within the article or from the corresponding author upon reasonable request.

## Supplementary Materials

The following supporting information can be downloaded at: https://www.mdpi.com/article/10.3390/biomedicines11071811/s1, Figure S1. H2O2 could activate the autophagy response in ECs. Figure S2. AAV vectors targeting the vascular endothelium exhibited effective expression in lung tissue. Figure S3. Verification of the MITF–GAS5–mir23 positive feedback loop. Figure S4. Western blot verification of miR-23 targeting DGKE. Figure S5. The GAS5–MITF axis integrates the Nrf2 antioxidant pathway to inhibit mitROS production. Figure S6. In vivo validation of the protective effect of the MITF–GAS5–miR-23 positive feedback loop to inhibit mitROS production.
