# Supplementary material for: An Autophagy-Associated MITF–GAS5–miR-23 Loop Attenuates Vascular Oxidative and Inflammatory Damage in Sepsis
Source: Biomedicines. 2023 Jun 24;11(7):1811. doi: 10.3390/biomedicines11071811 (PMC10376991; doi:10.3390/biomedicines11071811)
Supplement: Supplementary file 1 [file biomedicines-11-01811-s001.zip › biomedicines-2410584-supplementary.pdf]

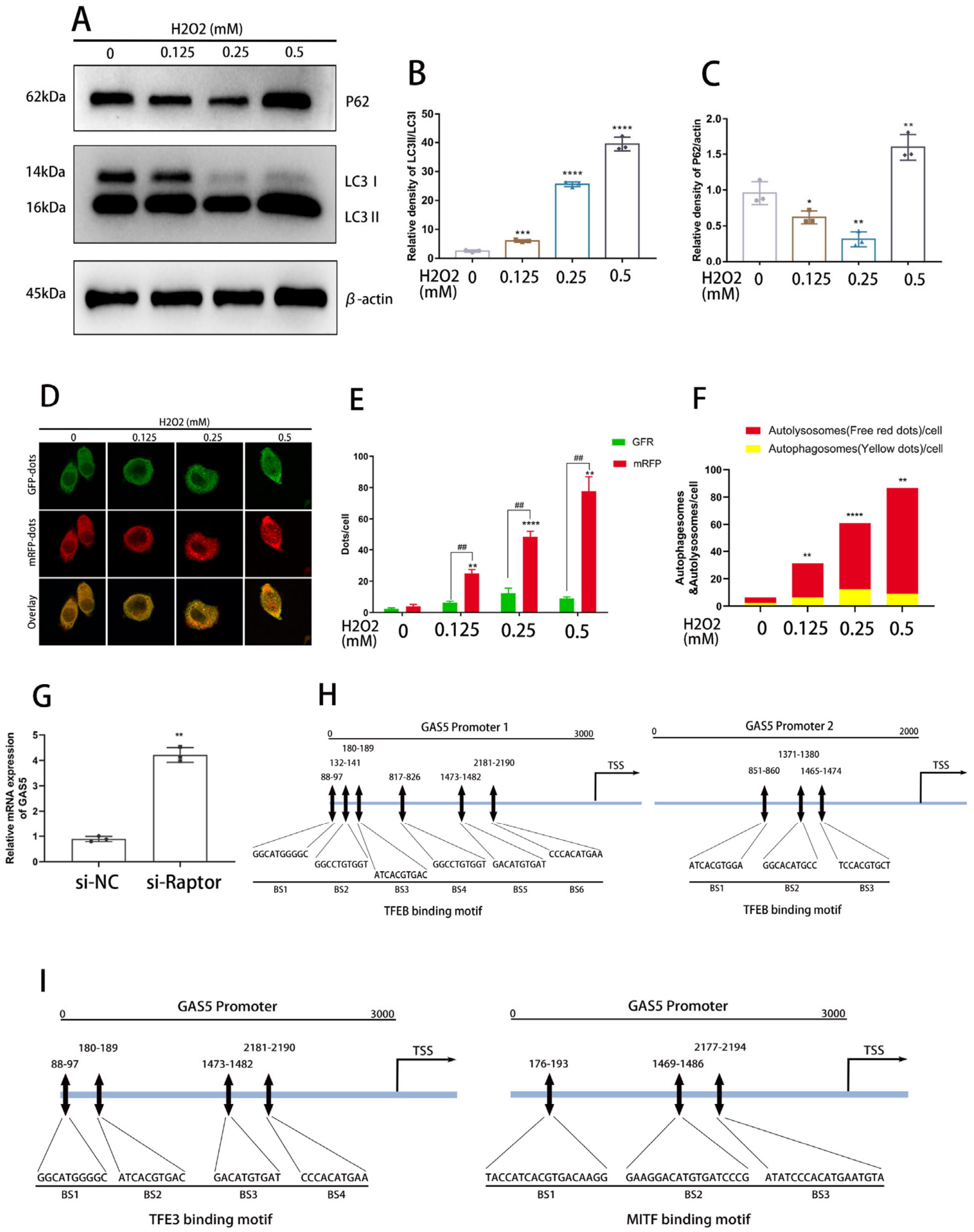

Figure S1 H2O2 could activate the autophagy response in ECs.

(A-C)Western blot analysis of LC3 conversion and P62 levels in HUVECs treated with H2O2. (D)HUVECs were treated with different concentrations of H2O2 for 24 h after mRFP-GFP-LC3 adenovirus transfection to assess autophagic flux. (E)The number of red and green fluorescent dots following treatment with H2O2 for 24 h at a gradient concentration. (F)The number of autophagosomes and autolysosomes after treatment with gradient concentrations of H2O2 for 24 h. (G) GAS5 expression in raptor siRNA-transfected HUVECs after 48h, analyzed by qPCR. (H) Bioinformatic analysis identifying TFEB-binding motifs within GAS5 promoter regions 1 and 2. (I) Bioinformatic analysis identified TFE3 and MITF binding motifs within the promoter of GAS5. *P<0.05, **P<0.01, ***P<0.001, ****P<0.0001.

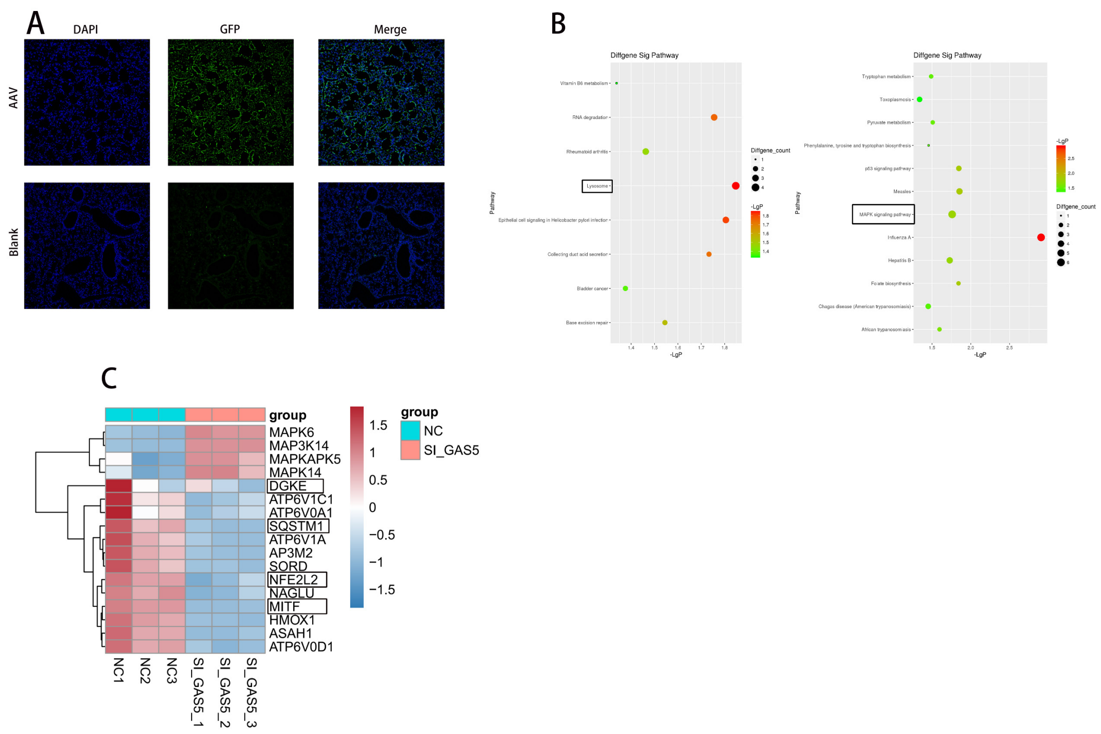

Figure S2 AAV vectors targeting the vascular endothelium exhibited effective expression in lung tissue.

(A)Use immunofluorescence to detect the expression of GFP in mice in the AAV group and the blank group to evaluate the infection effect. (B) KEGG analysis of significantly upregulated and downregulated pathways following GAS5 knockdown. (C) Heatmap of major differentially expressed genes after GAS5 knockdown.

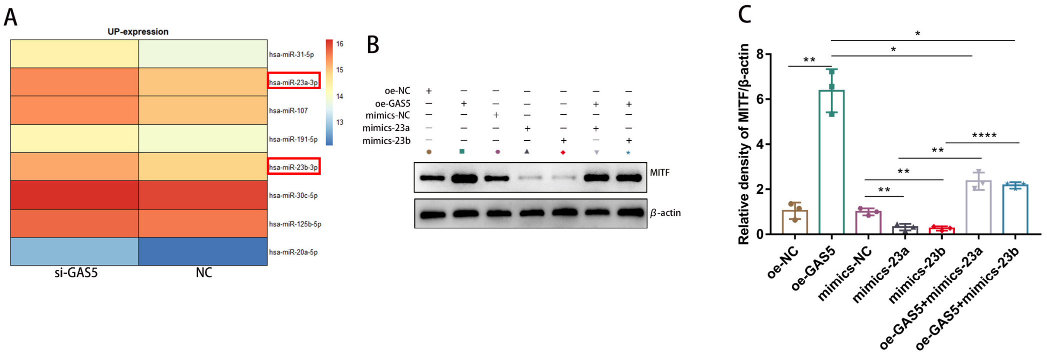

Figure S3 Verification of the MITF-GAS5-mir23 positive feedback loop.

(A)Heat map showed differential miRNA after GAS5 knockdown. (B)(C)After being transfected with miR-23a/b mimics or GAS5, MITF was determined by western blot. *P<0.05, **P<0.01, ***P<0.001, ****P<0.0001.

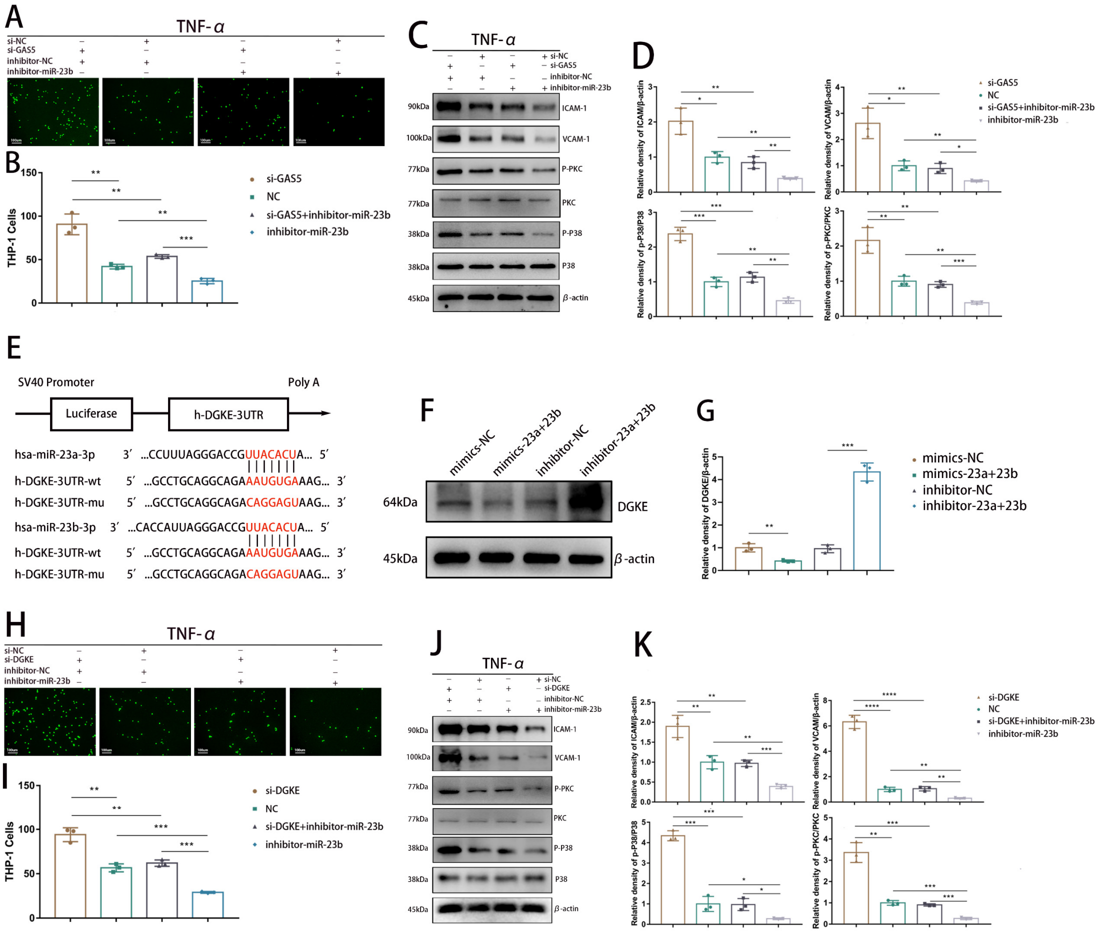

Figure S4 Western blot verification of miR-23 targeting DGKE.

(A-D) HUVECs in the control group, GAS5 knockdown group, miR-23b inhibitor group, and GAS5-KD+miR-23b-inhibitor group were treated with TNF-α. (A-B) A monocyte adhesion test was used to evaluate the adhesion ability of HUVECs to THP-1 monocytes under inflammation (A with quantification in B). (C-D) Western blot analysis was used to evaluate the expression levels of p38, p-p38, PKC, p-PKC, ICAM-1 and VCAM-1 in HUVECs (C with quantification in D). (E) The predicted binding site of miR-23a and miR-23b on the DGKE 3’ UTR. (F-G)Western blot analysis of DGKE levels in HUVECs treated with mir-23a+mir-23b mimics and inhibitors. (H-K) HUVECs in the control group, DGKE knockdown group, miR-23b inhibitor group, and DGKE-KD+miR-23b-inhibitor group were treated with TNF-α. (H-I) A monocyte adhesion test was used to evaluate the adhesion ability of HUVECs to THP-1 monocytes under inflammation (H with quantification in I). (J-K) Western blot analysis was used to evaluate the expression levels of p38, p-p38, PKC, p-PKC, ICAM-1 and VCAM-1 in HUVECs (J with quantification in K). *P<0.05, **P<0.01, ***P<0.001, ****P<0.0001.

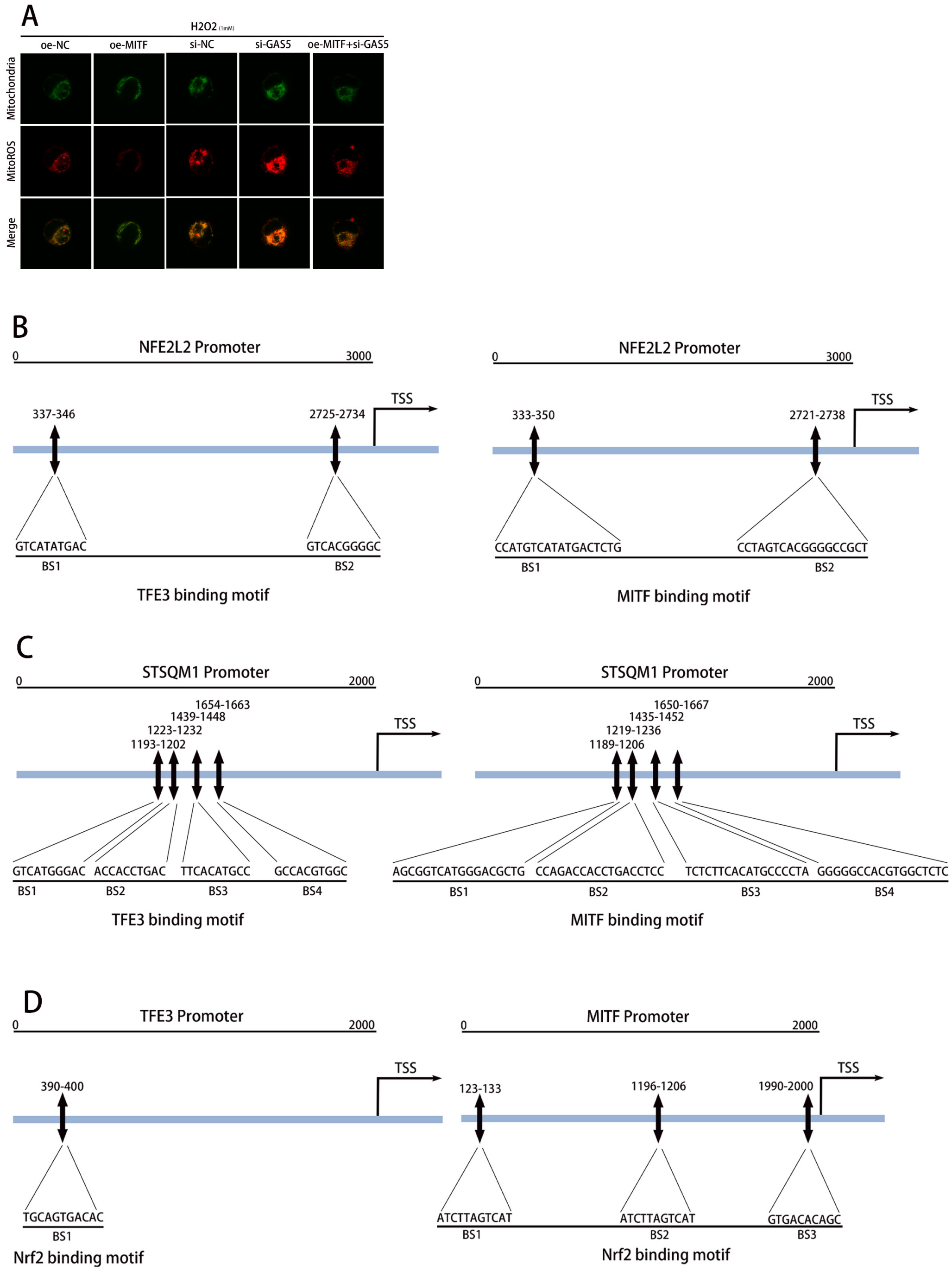

Figure S5 The GAS5-MITF axis integrates the Nrf2 antioxidant pathway to inhibit mitROS production.

(A)MitoSOXTM Red Mitochondrial Superoxide Indicator was used to measure mitROS in HUVECs. (B) Bioinformatic analysis identified TFE3-binding motifs and MITF-binding motifs within the promoter of the NFE2L2 gene. (C) Bioinformatic analysis identified TFE3-binding motifs and MITF-binding motifs within the promoter of the SQSTM1 gene. (D)Bioinformatic analysis identified Nrf2-binding motifs within the promoters of the TFE3 and MITF genes.

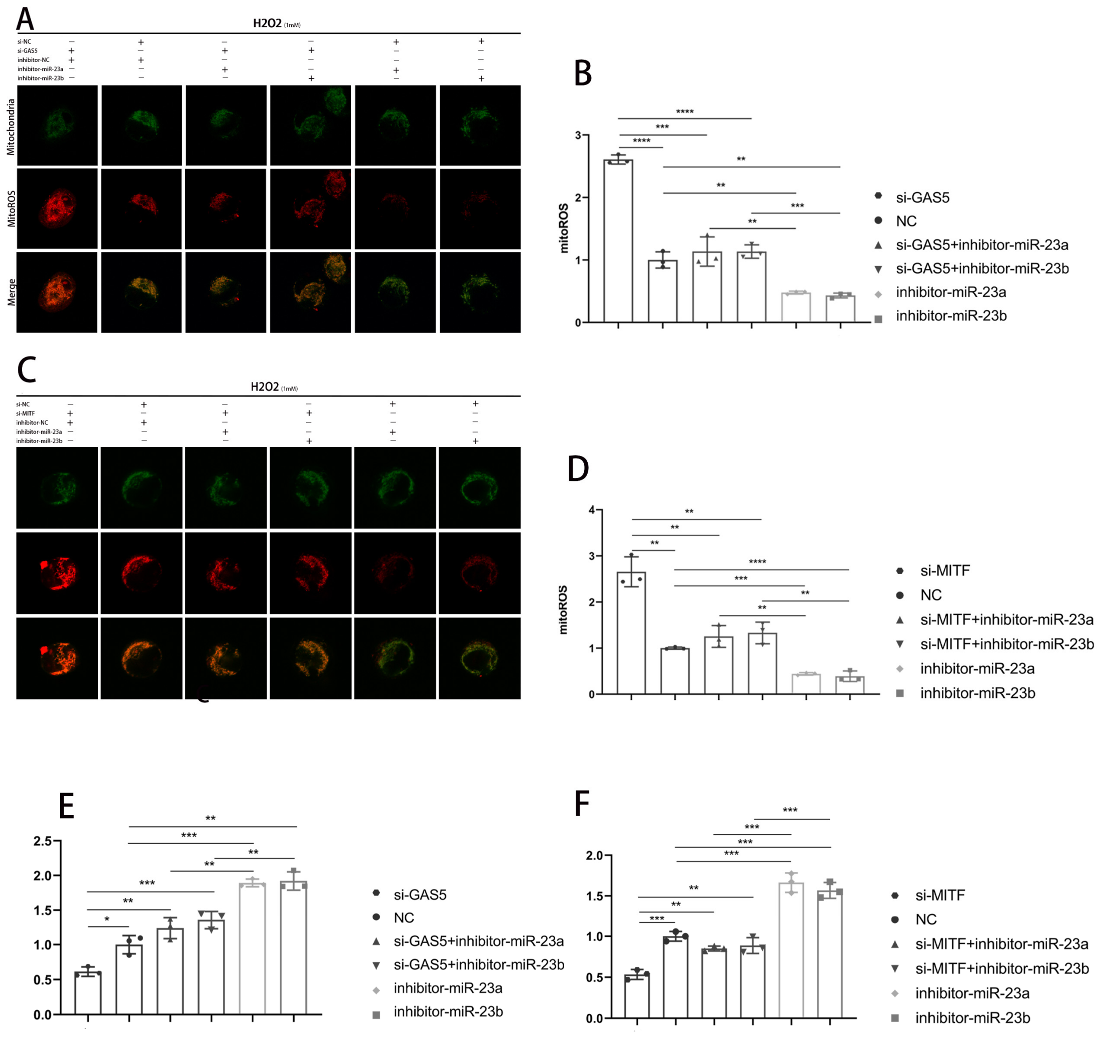

Figure S6 In vivo validation of the protective effect of the MITF-GAS5-miR-23 positive feedback loop to inhibit mitROS production.

(A-F) HUVECs in the control group, GAS5 knockdown group, miR-23a/b inhibitor group, GAS5-KD+miR-23a/b-inhibitor group, MITF knockdown group, and MITF-KD+miR-23a/b-inhibitor group were treated with H2O2. (A-D)MitoSOXTM Red Mitochondrial Superoxide Indicator was used to measure mitROS in HUVECs. (E)(F) The normalized transendothelial electrical resistance (TER) of HUVECs at 240 min after H2O2 treatment.
